# miR-4521-FAM129A axial regulation on ccRCC progression through TIMP-1/MMP2/MMP9 and MDM2/p53/Bcl2/Bax pathways

**DOI:** 10.1038/s41420-019-0167-5

**Published:** 2019-04-15

**Authors:** Xue Feng, Naimeng Yan, Weibin Sun, Shanliang Zheng, Sixiong Jiang, Jinxia Wang, Chunmei Guo, Lihong Hao, Yuxiang Tian, Shuqing Liu, Ming-Zhong Sun

**Affiliations:** 1 Department of Biotechnology, College of Basic Medical Sciences, Dalian Medical University, 116044 Dalian, China; 2 Department of Biochemistry, College of Basic Medical Sciences, Dalian Medical University, 116044 Dalian, China; 3 Department of Urology, The Second Affiliated Hospital, Dalian Medical University, 116027 Dalian, China; 4 Department of Anatomy, College of Basic Medical Sciences, Dalian Medical University, 116044 Dalian, China

## Abstract

Clear cell renal cell carcinoma (ccRCC) is the most aggressive RCC subtype with high metastasis, chemotherapy and radiotherapy resistance, and poor prognosis. This study attempted to establish the deregulations of miR-4521 and FAM129A together with their correlation to and mechanism of regulation of ccRCC development and progression. FAM129A acted as tumor promotor and miR-4521 acted as a suppressor in ccRCC. As measured in surgical tumorous tissues from ccRCC patients, FAM129A overexpression and miR-4521 deficiency together contributed to ccRCC progression by promoting advances in patients’ TNM stage and Fuhrman grade. Both the FAM129A knockdown and miR-4521 overexpression could reduce the in vitro migration and invasion abilities of renal cancer cells 786-O and ACHN, through the TIMP-1/MMP2/MMP9 pathway and could decrease their proliferation by promoting their apoptosis through the MDM2/p53/Bcl2/Bax pathway. By directly targeting the 3′-UTR domain of *FAM129A*, miR-4521 was negatively correlated with *FAM129A*/FAM129A levels in ccRCC progression and renal cancer cell malignancies. This work establishes the miR-4521-FAM129A axial regulation mechanism in ccRCC. Micro-4521 deficiency leads to *FAM129A*/FAM129A upregulation, which synergistically enhances the migration and invasion of renal cancer cells due to the induced decrease of TIMP-1 and increases of MMP2 and MMP9, and increases their growth through escaping apoptosis by suppressing p53 by way of upregulation of induced MDM2. The current work provides new clues to assist fundamental research into the diagnosis and treatment of ccRCC.

## Introduction

Renal cell carcinoma (RCC) comprises up to 85% of kidney cancer cases^1^. As one of the most common malignant urological tumors, RCC is characterized by a high mortality-to-incidence ratio and poor prognosis for late-stage patients^2–4^. Clear cell RCC (ccRCC), accounting for ~80% of RCCs, is the most aggressive RCC subtype. Accompanied by extremely high rates of local invasion, metastasis, and resistances to chemotherapy and radiotherapy, the 5-year survival rate of the over 30% of ccRCC patients with metastasis was below 20%^5–7^. The pathogenesis, diagnosis and treatment of ccRCC deserves more attention.

MicroRNAs (miRNAs) play important roles in the pathogenesis, development and prognosis of various major diseases by degrading mRNA or by inhibiting the translation processes of target genes by binding their 3′UTRs^8–12^. As a member of tRNA-derived small RNAs (tsRNAs), miR-4521 is involved in breast cancer, chronic lymphocytic leukemia (CLL), lung cancer, pancreatic ductal adenocarcinoma (PDAC) and esophageal adenocarcinoma^12–15^. Its deregulation in MCF-7 cells is accompanied by hypoxic hypoxia induction^13^ and in enhanced malignancy of CLL and lung cancer^14^. It was one of 11 joint miRNAs implicated in the prognosis of PDAC patients with pancreaticoduodenectomy^12^. A higher miR-4521/miR-340-5p ratio was implicated in better disease-free survival of esophageal adenocarcinoma patients with neoadjuvant chemoradiotherapy and esophagectomy^15^. Except for the finding that miR-4521 was downregulated in the sunitinib-resistant ACHN and RCC23 cell lines, SR-ACHN and SR-RCC23^16^, no study has addressed its role or regulation mechanism in ccRCC. In this study miR-4521 acted as a tumor suppresser in ccRCC. Its deficiency negatively correlated with FAM129A upregulation and prompted clinical development and progression of ccRCC. It mediated ccRCC carcinogenesis by affecting cancer cells’ malignant invasiveness via the TIMP-1/MMP2/MMP9 and MDM2/p53/Bcl2/Bax pathways.

The member A of family with sequence similarity 129 (FAM129A), also known as *Niban*, was originally identified in Eker rats with hereditary renal carcinoma induced by tuberous sclerosis 2 gene mutation (Tsc2)^17^. It is commonly overexpressed in patients with thyroid cancer^18–20^, head and neck squamous cell carcinoma (HNSCC)^21^ and sporadic renal carcinomas^22^. Although highly expressed in early stages of cancer development and remaining overexpressed throughout cancer progression, its function and mechanism of action remain unclear. FAM129A was detected in sporadic RCCs including clear cell, granular cell and spindle cell carcinomas. Its common expression might be an indicator for renal carcinogenesis^17,22^. FAM129A has been linked to renal interstitial fibrosis by increasing renal tubular cell apoptosis^23^; however, its function and mechanism in ccRCC remain unclear.

Current work shows FAM129A is a promotor and miR-4521 is a suppressor in ccRCC. FAM129A overexpression positively correlates with advances in TNM stage and Fuhrman grade of ccRCC patients. miR-4521 deficiency contributed to enhanced TNM stage and Fuhrman grade of ccRCC. FAM129A knockdown, miR-4521 overexpression with its induced FAM129A downregulation decreased the proliferation, migration and invasion, and increased the apoptosis of renal carcinoma cells through the TIMP-1/MMP2/MMP9 and MDM2/p53/Bcl2/Bax pathways.

## Results

### miR-4521 deficiency links to advances in TNM stage and Fuhrman grade of ccRCC

**Fig. 1:**
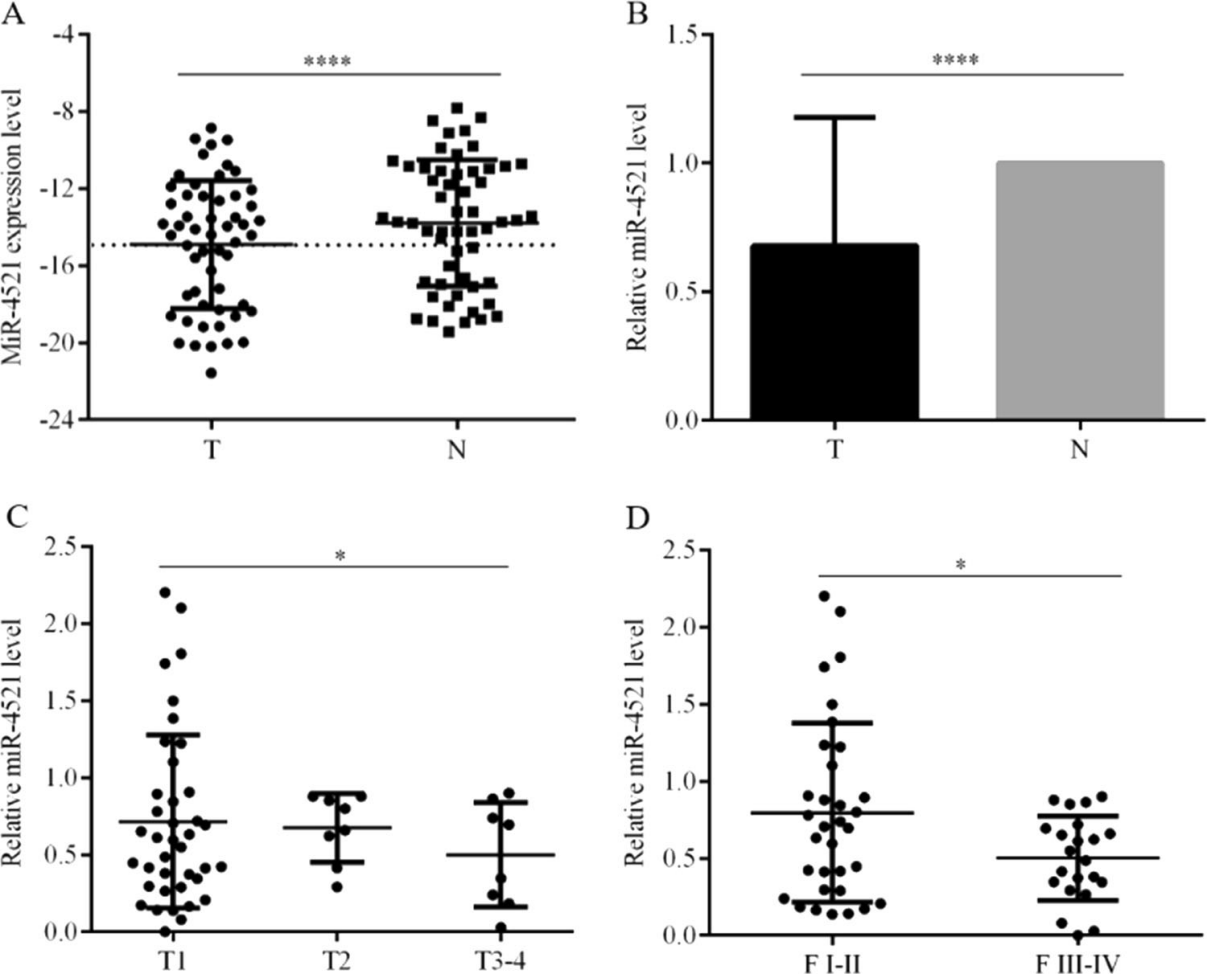
Expression of miR-4521 in ccRCC surgical tumorous tissues and its link with TNM stage and Fuhrman grade of patients. **a** miR-4521 expression levels were significantly downregulated in ccRCC cancer tissues compared with paracancerous nontumor renal tissues. **b** The downregulation efficiency of miR-4521 in tissue samples is shown. Correlations between miR-4521 expression level and the TNM stage (**c**) and Fuhrman grades (**d**) of ccRCC are shown, respectively. ccRCC clear cell renal cell carcinoma. *P < 0.05, ****0.0001

miR-4521 expression was decreased in patients’ tumorous tissues (Fig. 1a). Compared with paired paracancerous nontumor tissues, miR-4521 levels in tumorous tissues from 55 ccRCC patients decreased by 32.2% (*P* < 0.0001, Fig. 1b). Among patients’ clinicopathological parameters, miR-4521 deficiency was associated with patients’ TNM stage (Fig. 1c, *P* = 0.017) and Fuhrman grade (Fig. 1d, *P* = 0.029, Table 1), but was unrelated to patients’ age (*P* = 0.077), gender (*P* *=* 0.094) and tumor location (kidney size, *P* = 0.195, Table 1). Compared with T1 patients, miR-4521 levels in tumorous tissues from ccRCC patients in T2 and T3-4 decreased by 5.7% and 30.2%, respectively (Fig. 1c, Table 1, *P* = 0.017). Compared with Fuhrman I-II patients, miR-4521 decreased 37.2% in tumorous tissues from Fuhrman III-IV patients (Fig. 1d, Table 1, *P* = 0.029). miR-4521 deficiency contributes to ccRCC development and progression.

**Table 1:** Clinical association of miR-4521 and FAM129A level changes with ccRCC

Characteristics	mRNA and protein expression level change and clinical significance							
	No. of patient (55 in total)	Relative miR-4521 level^a^	*P* value	Relative *FAM129A* level^a^	*P* value	No. of patient (37 in total)	Relative FAM129A level	*P* value
Age, years								
≤60	34	0.772	0.077	3.476	0674	23	2.834	0.429
>60	21	0.527		3.056		14	3.661	
Gender								
Male	32	0.774	0.094	3.316	0.999	23	3.846	0.072
Female	23	0.545		3.315		14	1.999	
Tumor position								
Left kidney	33	0.750	0.195	3.312	0.994	25	3.263	0.742
Right kidney	22	0.571		3.320		12	2.905	
Fuhrman grade								
I-II^b^	33	0.797	0.029	2.522	0.041	20	2.154	0.028
III-IV^c^	22	0.500		4.505		17	4.315	
TNM stage								
T1	39	0.716		2.658		22	2.205	
T2^d^	8	0.675	0.017	2.980	0.007	7	3.393	0.024
T3-4^e^	8	0.499		6.856		8	5.523	

*ccRCC* clear cell renal cell carcinoma

^a^Refers to the ratio of miR-4521 and *FAM129A* mRNA expression level in tumorous tissues divided by that in paracancerous tissue

^b^Four patients in Fuhrman grade I-II and six patients in grade II-III were considered together with 23 patients in grade II

^c^One patient in Fuhrman grade IV and one patient in grade III-IV were considered together with 20 patients in grade III

^d^One patient at stage T2-3 was considered together with seven ccRCC patients at stage T2

^e^One patient at stage T4 was considered together with seven ccRCC patients at stage T3

### FAM129A upregulation enhances advances in ccRCC TNM stage and Fuhrman grade

**Fig. 2:**
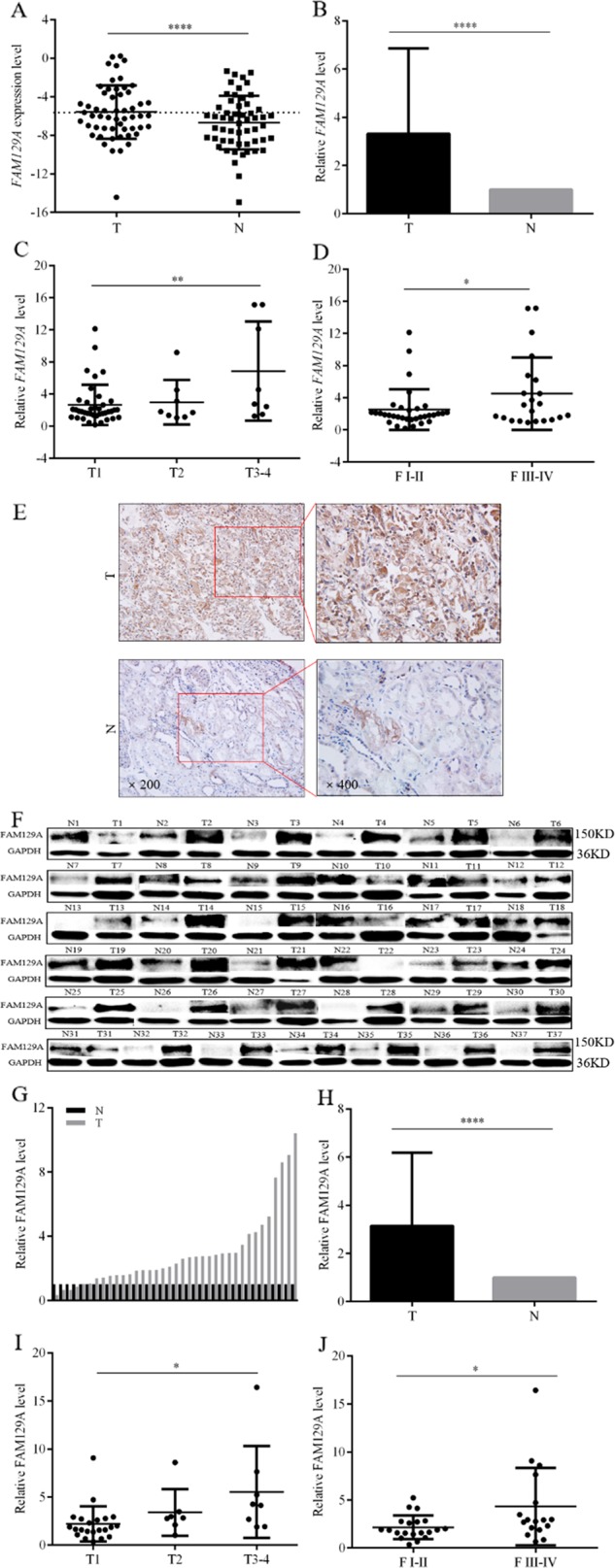
Expression of FAM129A in ccRCC patients’ tumorous tissues and associated with TNM stage and Fuhrman grade. **a** Upregulation of FAM129A mRNA levels in ccRCC cancer tissues; normal tissues served as controls. **b** The upregulation efficiency of FAM129A in tissue samples. **c**, **d** Correlations between the FAM129A mRNA expression level and the TNM stage and Fuhrman grades of ccRCC. **e** Representative images of FAM129A IHC in ccRCC cancer tissues and normal tissues. **f**, **g** Western blots of FAM129A showed alterations in protein levels consistent with variations in mRNA levels in clinical samples. **h** Protein expression of FAM129A is high compared with normal tissues. Correlations between FAM129A protein levels and the TNM stage (**i**) and Fuhrman grades (**j**) of ccRCC are shown. ccRCC clear cell renal cell carcinoma, IHC immunohistochemistry. *P values < 0.05, **0.01, ****0.0001

At the mRNA level (Fig. 2a), compared with paracancerous nontumor renal tissues, *FAM129A* levels in tumorous tissues increased by 231.5% (*P* < 0.0001, Fig. 2b). *FAM129A* upregulation positively correlated with advances in TNM stage (Fig. 2c) and Fuhrman grade (Fig. 2d) (Table 1). *FAM129A* levels in ccRCC patients with T2 and T3-4 stages increased by 12.1% and 157.9% more than T1 patients (Fig. 2c, Table 1, *P* = 0.007). Compared with Fuhrman grade I-II patients, *FAM129A* increased by 78.6% for Fuhrman grade III-IV patients (Fig. 2d, Table 1, *P* = 0.041).

IHC showed FAM129A is predominantly localized in the cytoplasm (Fig. 2e). Out of 30 ccRCC patients’ samples, 0, 2, 10 and 18 tumorous tissues and 21, 6, 2 and 1 paracancerous tissues showed the immunoreactivities of −, +, ++ and +++. FAM129A was 70% (*P* = 0.0001) higher in tumorous tissues. FAM129A-positive detection rates of tumorous tissues with ++ and +++ were 5 (10/2) and 18 (18/1) fold of those of nontumor tissues. Its upregulation was associated with patients’ TNM stage (*P* = 0.015) and showed a high correlation with patients’ Fuhrman grade (*P* = 0.170, Table 2). WB results (Fig. 2f) showed, compared with paired paracancerous tissues, FAM129A was upregulated in 31 cases, unchanged in 3 cases, and decreased in 3 cases of the 37 available from 55 patients’ tumorous tissues (Fig. 2g). FAM129A global levels increased by 214.7% (Fig. 2h, *P* < 0.0001) in tumorous tissues. Its protein upregulation was linked to advances in TNM stage (Fig. 2i, *P* = 0.024) and Fuhrman grade (Fig. 2j, *P* = 0.028). *FAM129A*/FAM129A overexpression contributes to ccRCC development and progression.

**Table 2:** IHC assay of clinical association of FAM129A level change with ccRCC

Characteristics	No. of specimen (30 in total)	FAM129A immunoreactivity degree				*P* value
		−	+	++	+++	
Age, years						
≤60	18	0	1	6	11	0.8
>60	12	0	1	4	7	
Gender						
Male	22	0	1	8	13	1
Female	8	0	1	2	5	
Tumor position						
Left kidney	22	0	1	8	13	1
Right kidney	8	0	1	2	5	
Fuhrman grade						
I-II	14	0	0	4	10	0.17
III-IV	16	0	2	6	8	
TNM stage						
T1	15	0	1	8	6	0.01
T2	8	0	1	1	6	
T3-4	7	0	0	1	6	

*IHC* immunohistochemistry, *ccRCC* clear cell renal cell carcinoma

### miR-4521 negatively correlates with FAM129A in ccRCC tumorous tissues

**Fig. 3:**
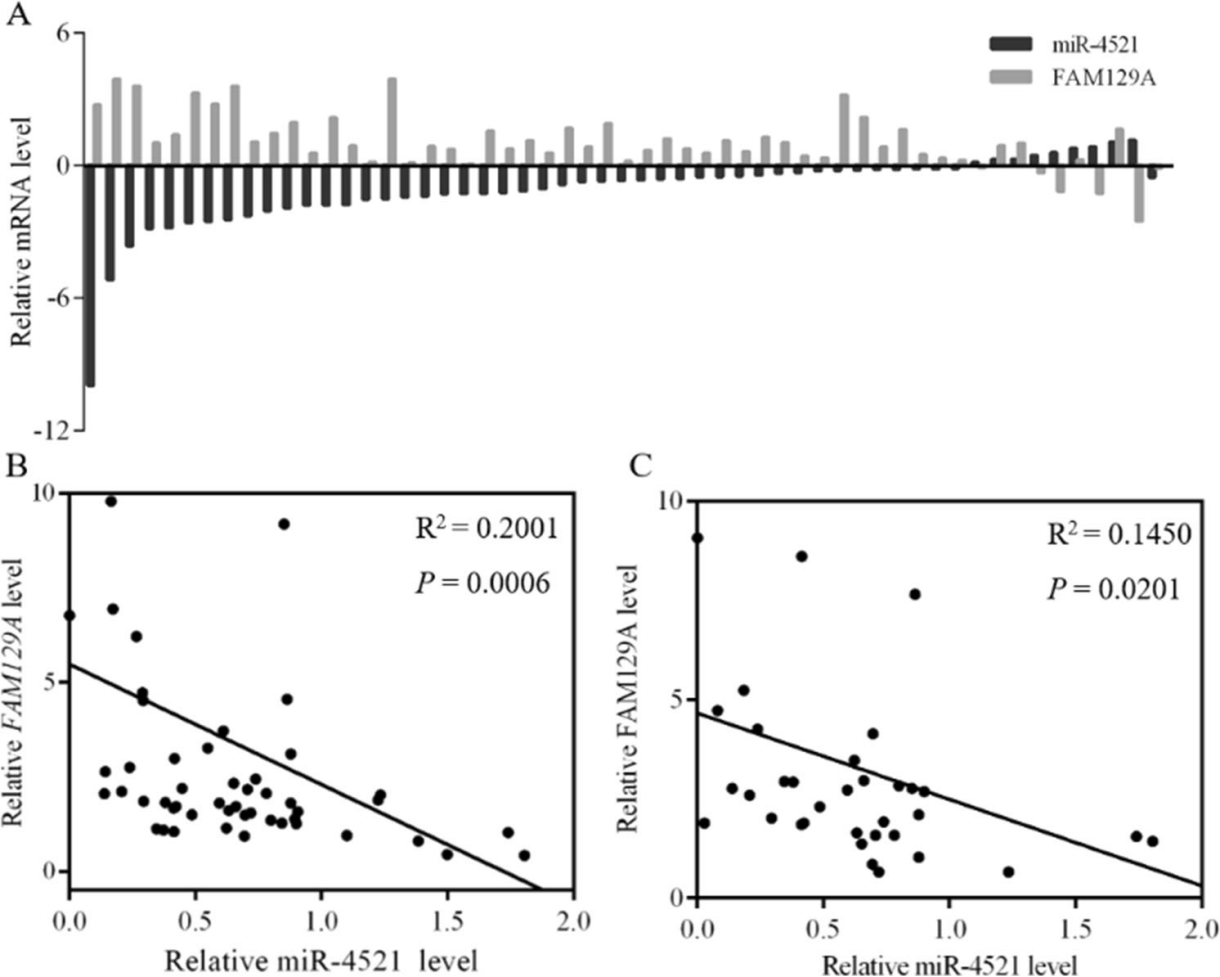
miR-4521 deficiency is negatively correlated with FAM129A upregulation in clinical tumorous tissues of ccRCC patients. **a** 45 cases in 55 samples showed high mRNA expression of FAM129A when miR-4521 expression was low. Inverse correlations of FAM129A mRNA (**b**) and protein (**c**) levels with miR-4521 levels in ccRCC tissue are shown. ccRCC clear cell renal cell carcinoma

*FAM129A* mRNA upregulation was detected in most cases (45/55) where patients’ tumorous tissues were accompanied by miR-4521 downexpression (Fig. 3a). Its upregulation inversely correlated with miR-4521 downregulation (*P* = 0.0006, Fig. 3b). Consistent with this, FAM129A protein upregulation (Fig. 2f) also inversely correlated with miR-4521 downregulation (*P* = 0.020, Fig. 3c). Hence, the correlation and regulation mechanism between FAM129A and miR-4521 was investigated and established.

### miR-4521 downregulates and FAM129A overexpresses in RCC cells

**Fig. 4:**
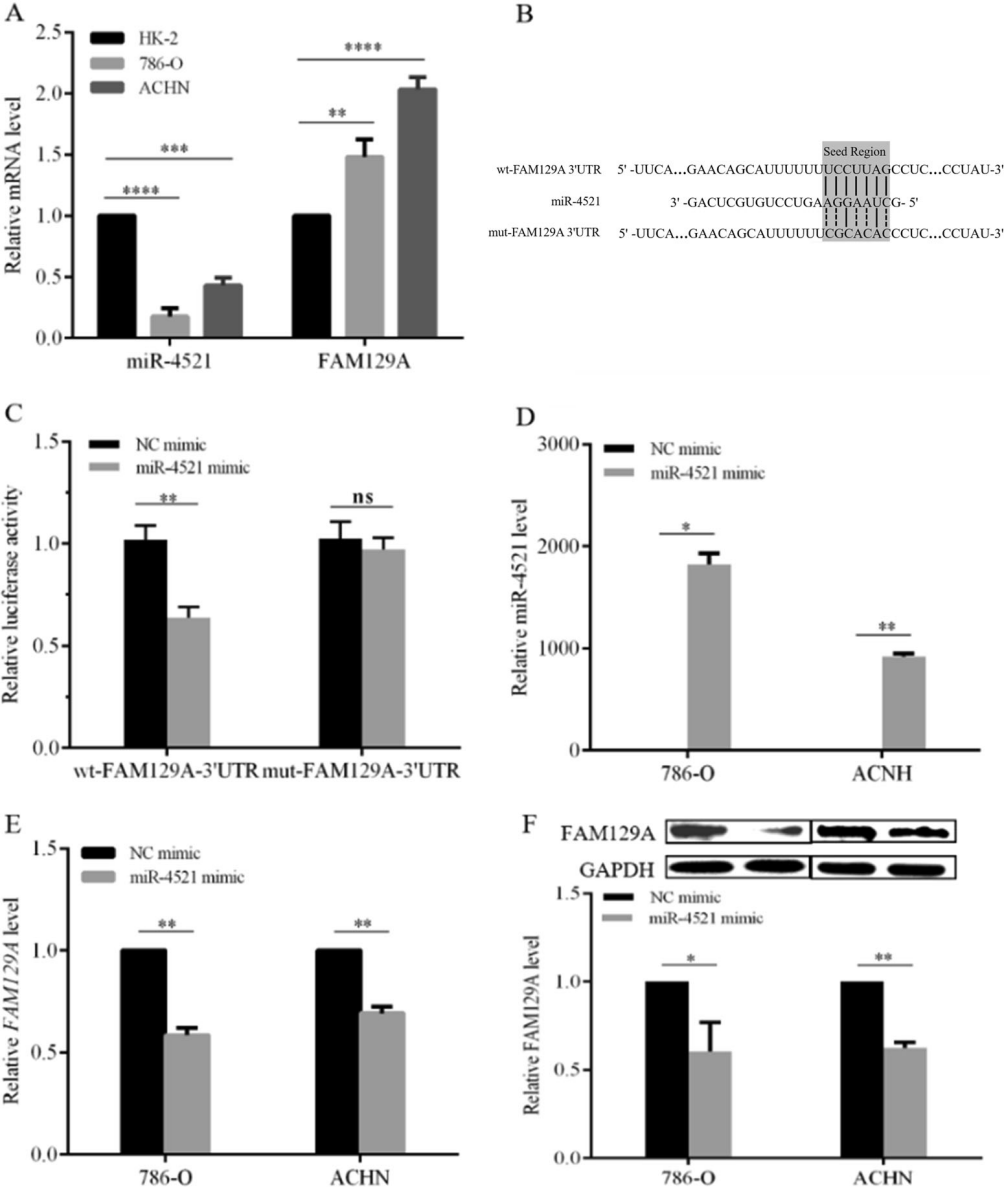
miR-4521 downexpression accompanied with FAM129A overexpression in RCC cell lines through binding with the latter’s 3′-UTR domain. **a** miR-4521 and FAM129A expressions in RCC cell lines (786-O and ACHN) compared with that in HK-2. **b** Sequence alignment of the FAM129A 3′UTR with wild-type (WT) versus mutant (MUT) potential miR-4521 targeting sites. **c** Dual-luciferase reporter assays showed decreased reporter activity after transfection of the wild-type FAM129A 3′UTR reporter construct in 786-O cells overexpressing miR-4521. The FAM129A 3′UTR MUT and control constructs showed no effect on reporter activity. **d** Alteration of the miR-4521 expression levels of 786-O and ACHN cells 24 h after transfection with the miR-4521 mimics or NC mimics. mRNA (**e**) and protein (**f**) levels of FAM129A were examined by qRT-PCR and Western in 786-O and ACHN cells that were transfected with miR-4521/NC mimics. *P values < 0.05, **0.01, ***0.001, ****0.0001

Compared with HK-2, a human proximal tubule epithelial cell line, miR-4521 reduced by ~82.4% (*P* *<* 0.0001) and 56.9% (*P* *=* 0.0001, Fig. 4a) and *FAM129A* increased by ~48.1% (*P* = 0.0041) and 103.3% (*P* *<* 0.0001, Fig. 4a) in RCC cell lines 786-O and ACHN. Their inversely correlated (*P* = 0.005) expressions showed the same trend as patients’ specimens. Thus 786-O and ACHN are appropriate cell models for investigating the function and regulation mechanism of miR-4521 and FAM129A in ccRCC.

miRDB (http://mirdb.org/miRDB) and TargetScan (http://www.targetscan.org) showed the sequence AGGAAUC of miR-4521 (Fig. 4b) complementarily binds with UCCUUAG (Fig. 4b) at sites 3232−3238 of *FAM129A*. Dual-luciferase reporter assays combined with site mutation confirmed their direct targeting in 786-O cells. The plasmids of the 211 bp fragment of *FAM129A* 3′UTR containing wild-type (WT) and mutant (MUT, UCCUUAG mutated to CGCACAC) sequences were inserted into a psiCHECK™2.0 plasmid, named as wt-FAM129A-3′UTR and mut-FAM129A-3′UTR (Fig. 4b). The recombinant plasmids were then cotransfected into 786‐O with miR-4521 mimic and NC mimic. Compared with the NC group, luciferase activity decreased by 38.3 ± 6.9% (*P* = 0.0015) in 786-O cells cotransfected with wt-FAM129A-3′UTR and miR-4521 mimic, while luciferase activity did not change in 786-O cells cotransfected with mut-FAM129A-3′UTR and miR-4521 mimic (*P* = 0.3122, Fig. 4c). FAM129A is a direct downstream target of miR-4521.

miR-4521 deregulation affected FAM129A expression. Compared with NC cells, miR-4521 increased 1820- (*P* = 0.01) and 920-fold (*P* = 0.0005, Fig. 4d) in miR-4521-mimic-transfected 786-O and ACHN cells. *FAM129A* mRNA levels decreased by 41.5% (*P* = 0.003) and 30.8% (*P* = 0.005, Fig. 4e) and FAM129A protein levels decreased by 36.2% (*P* = 0.01) and 37.6% (*P* = 0.002, Fig. 4f). miR4521 retroregulates FAM129A. Its suppression contributes to elevated FAM129A in enhanced RCC malignancy.

### FAM129A knockdown reduces in vitro malignant properties of 786-O and ACHN

**Fig. 5:**
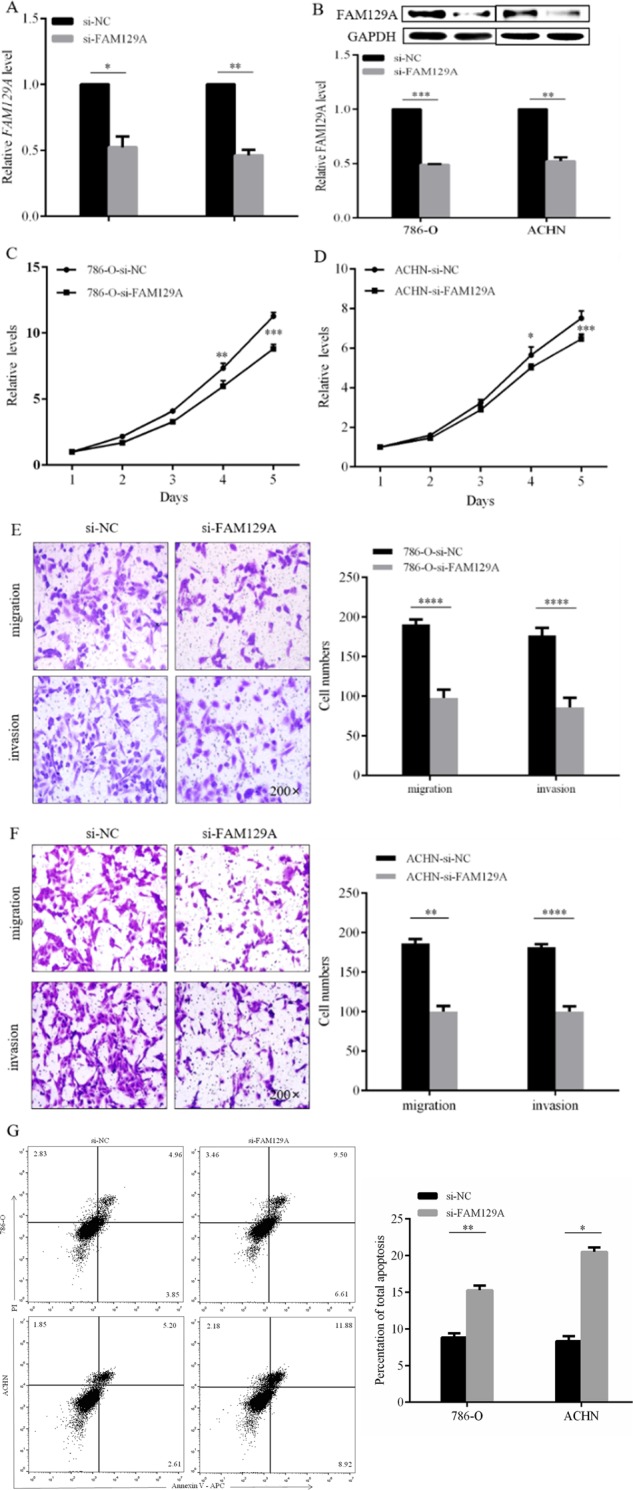
FAM129A knockdown reduces the in vitro malignant behaviors of 786-O and ACHN cells. mRNA (**a**) and protein (**b**) levels of FAM129A were examined by qRT-PCR and western blot in 786-O and ACHN cells that were transfected with si-NC /si-FAM129A. MTT assays revealed that transfection of the siRNA of FAM129A can remarkably attenuate the proliferation of 786-O (**c**) and ACHN (**d**) cells compared with siNC. Representative photographs are shown of transwell assays of 786-O (**e**) and ACHN (**f**) cells identifying FAM129A as oncogenic. **g** Flow cytometry was performed to reveal that FAM129A downregulation largely induces the cell apoptosis of 786-O and ACHN cells. Each experiment was performed in triplicate. *P *<* 0.05; **P *<* 0.01; ***P *<* 0.001; ****P < 0.0001

Compared with the irrelevant siRNA (si-NC)-transfected cells, the mRNA and protein levels of FAM129A decreased by 37.6% (*P* = 0.0145) and 53.8% (*P* = 0.003, Fig. 5a), and by 51.0% (*P* = 0.001) and 47.7% (*P* = 0.0023, Fig. 5b) in si-FAM129A-transfected 786-O and ACHN cells. FAM129A knockdown reduced their proliferation, migration and invasion capacities, and enhanced their apoptosis (Fig. 5).

The proliferation rates of si-FAM129A-transfected 786-O and ACHN decreased by 19.0% (*P* = 0.008) and 12.6% (*P* = 0.01, Fig. 5c) at 96 h, and by 22.2% (*P* = 0.0001) and 18.7% (*P* = 0.0006, Fig. 5d) at 120 h. Compared with si-NC-transfected cells, the migration and invasion capacities of si-FAM129A-transfected 786-O were reduced by 48.8% (*P* = 0.0001) and 51.7% (*P* < 0.0001, Fig. 5e), and si-FAM129A-transfected ACHN were decreased by 46.2% (*P* = 0.0055) and 45.1% (*P* < 0.0001, Fig. 5f). Compared with the apoptotic percentages of si-NC-transfected cells, the apoptotic percentages of si-FAM129A-transfected 786-O and ACHN increased by 42.5% (*P* = 0.004) and 59.5% (*P* = 0.04) to 15.3 ± 0.5% and 20.5 ± 0.4%, respectively (Fig. 5g). The fact that FAM129A knockdown antagonized malignant behaviors of RCC cells supported its overexpression in ccRCC clinical progression.

### miR-4521 overexpression decreases in vitro malignant properties of 786-O and ACHN via downregulating FAM129A

**Fig. 6:**
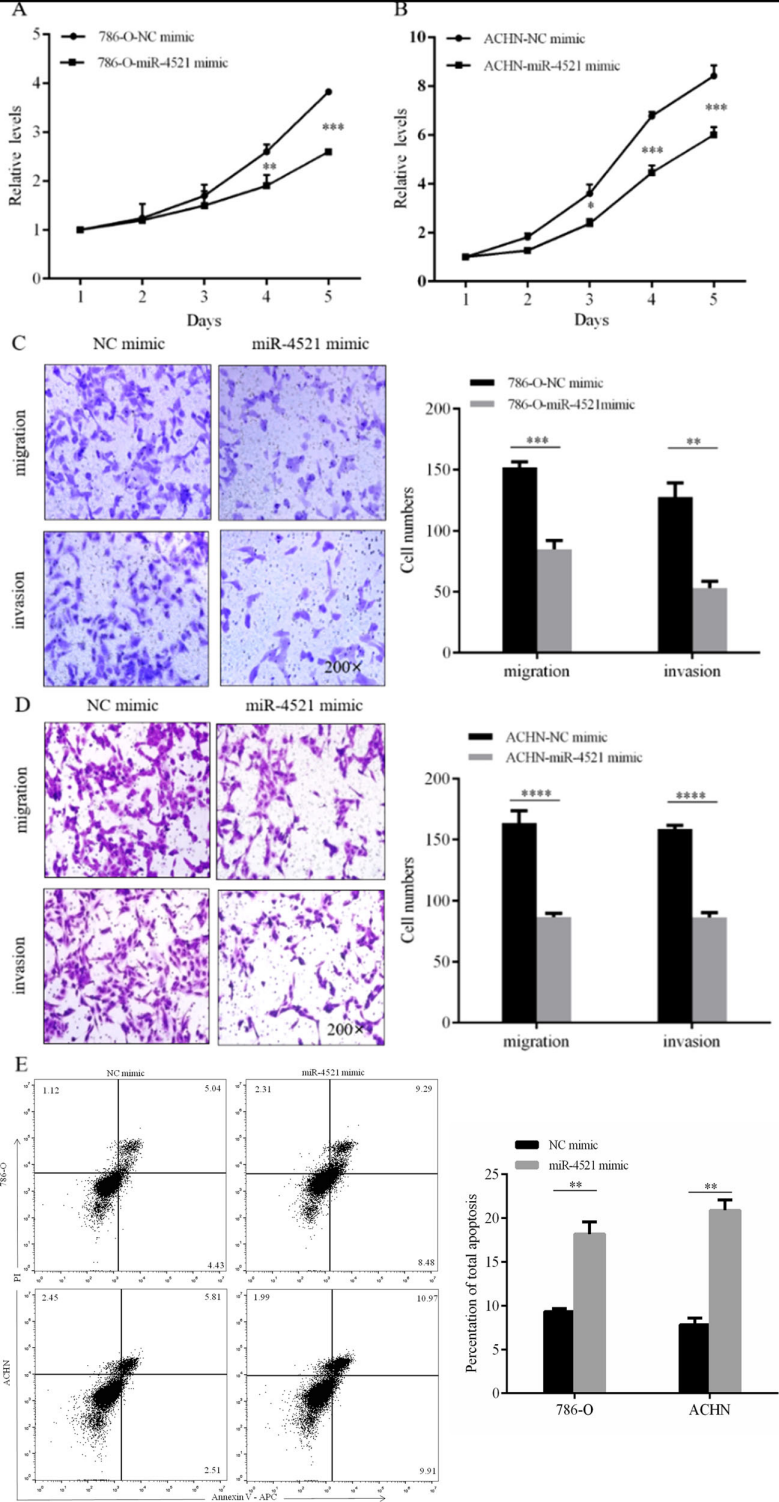
The influence of miR-4521 overexpression in vitro malignant properties of 786-O and ACHN cells. MTT assays showed that transfection of the miR-4521 mimic can significantly attenuate the proliferation velocity of 786-O (**a**) and ACHN (**b**) cells. Representative photographs are shown of migration and invasion assays of 786-O (**c**) and ACHN (**d**) cells to determine the tumor suppressor function of miR-4521. **e** Flow cytometry was performed to reveal that the miR-4521 mimic induced the cell apoptosis of 786-O and ACHN cells. *P values < 0.05, **0.01, ***0.001, ****0.0001

miR-4521 overexpression retroregulated FAM129A, which synergically decreased the malignant properties of 786-O and ACHN cells. First, miR-4521 overexpression reduced their proliferation. Compared with the NC group cells, the proliferation of miR-4521-mimic-transfected 786-O decreased by 28.8% (*P* = 0.005) and 32.1% (*P* = 0.0001, Fig. 6a) at 96 and 120 h, respectively and the proliferation of miR-4521-transfected ACHN decreased by 34.3% (*P* = 0.02), 37.4% (*P* = 0.0001) and 28.7% (*P* = 0.0001) at 72, 96 and 120 h, respectively (Fig. 6b). Second, miR-4521 overexpression decreased cellular migration and invasion. The migration and invasion abilities of 786-O and ACHN cells decreased by 50.0% (*P* = 0.0007) and 58.6% (*P* = 0.008, Fig. 6c), and by 47.0% (*P* = 0.0001) and 46.2% (*P* = 0.0001, Fig. 6d), respectively. Moreover, miR-4521 overexpression enhanced cellular apoptosis. Compared with the NC-mimic-transfected group cells, the apoptotic percentages of miR-4521-mimic-transfected 786-O and ACHN reached 18.2 ± 0.8% and 20.9 ± 0.8% increasing by 48.9% (*P* = 0.008) and 62.7% (*P* = 0.005, Fig. 6e) following miR-4521 overexpression.

### FAM129A mediates 786-O malignant behaviors via TIMP-1/MMP2/MMP9 and MDM2/p53/Bcl2/Bax

**Fig. 7:**
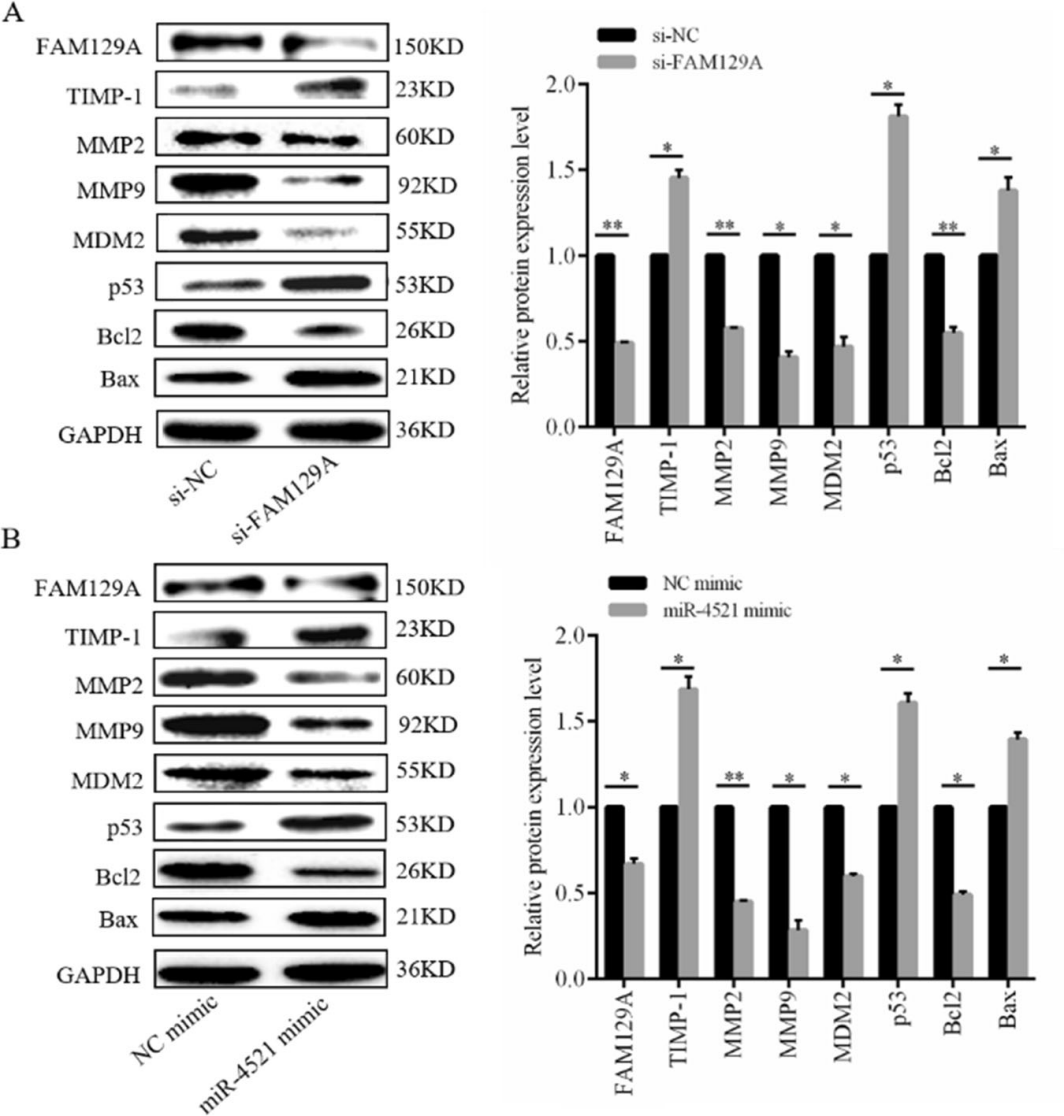
FAM129A and miR-4521 mediate the malignant behaviors of via TIMP-1/MMP2/MMP9 and MDM2/p53/Bcl2/Bax pathways in 786-O cells. The protein levels of FAM129A, TIMP-1, MMP2, MMP9, MDM2, p53, Bcl2 and Bax were examined using western blot analysis in 786-O cells treated with si-NC /si-FAM129A (**a**) or NC mimic/miR-4521 mimic (**b**). GAPDH was used as an internal control. *P < 0.05, **P < 0.01

Changes in TIMP-1, MMP2, MMP9, MDM2, p53, Bcl2 and Bax levels in 786-O cells following FAM129A knockdown were determined by WB. Compared with si-NC-transfected cells, MMP2, MMP9, MDM2 and Bcl2 levels decreased by 42.4% (*P* = 0.004), 49.2% (*P* = 0.02), 53.1% (*P* = 0.04) and 45% (*P* = 0.03), and TIMP-1, p53 and Bax levels increased by 45.3% (*P* = 0.04), 81.1% (*P* = 0.03) and 37.8% (*P* = 0.04) in si-FAM129A-transfected 786-O cells (Fig. 7a). FAM129A influences 786-O malignancy via TIMP-1/MMP2/MMP9 and MDM2/p53/Bcl2/Bax.

### miR-4521 deregulation mediates 786-O malignant behaviors via TIMP-1/MMP2/MMP9 and MDM2/p53/Bcl2/Bax

miR-4521 overexpression reduced FAM129A expression and malignant properties of 786-O and ACHN cells. Its deregulation should be associated with TIMP-1/MMP2/MMP9 and MDM2/p53/Bcl2/Bax, opposite to the trend induced by the FAM129A change. Compared with the NC group cells, WB results (Fig. 7b) indicated MMP2, MMP9, MDM2 and Bcl2 levels in miR-4521-mimic-transfected 786-O decreased by 55.1% (*P* = 0.006),72.5% (*P* = 0.03), 40.2% (*P* = 0.01) and 50.9%, respectively (*P* = 0.01), and TIMP-1, p53 and Bax levels increased by 68.4% (*P* = 0.04), 60.6% (*P* = 0.03) and 39.5%, respectively (*P* = 0.04). miR-4521 mediates RCC malignancy through direct interaction with FAM129A via TIMP-1/MMP2/MMP9 and MDM2/p53/Bcl2/Bax.

## Discussion

As the highest mortality urological malignancy^24,25^, ccRCC accounts for ~80% of RCC incidence^4^. The prognosis for cases of advanced ccRCC is poor^26^. A 5-year survival rate of the over 30% of patients with metastatic ccRCC is below 20%^5,6^. Better understanding on the pathogenesis, diagnosis and treatment of ccRCC^7^ is urgently needed.

Binding to 3′UTR regions of targeting genes, miRNAs cause degradation of mRNA or inhibit their translation processes. miRNAs play important roles in a variety of human major diseases including cancer^9–12^. miR-4521 dysexpression is involved in some cancers. Its deficiency is a contributing factor in the development and progression of CLL and lung cancer^14^. It was one of 11 miRNAs found to be prognostic indicators for PDAC patients^12^. The miR-4521/miR-340-5p ratio was regarded as a prediction factor for disease-free survival of esophageal adenocarcinoma patients^15^. miR-4521 has been linked to drug-resistance of RCC cells. In sunitinib-resistant ACHN and RCC23 cell lines, its expression level decreased^16^. Some miRNAs were reported as sensitive and specific indicators for the pathologic stage, recurrence, metastasis and survival of ccRCC patients^27^. The role and mechanism of action of miR-4521 in ccRCC has not been reported.

Our current work shows miR-4521 is a suppressor in ccRCC progression, interacting with FAM129A via the TIMP-1/MMP2/MMP9 and MDM2/p53/Bcl2/Bax pathways. miR-4521 downregulation was inversely correlated with the TNM stage and Fuhrman grade (Fig. 1, Table 1) of ccRCC patients. miR-4521 was also downexpressed in RCC cell lines 786-O and ACHN compared with HK-2 cells (Fig. 4a). 786-O and ACHN are appropriate cell models for studying the role and cellular regulation mechanism of miR-4521 in ccRCC malignancy. Since it is downregulated in both ccRCC patients’ tumorous tissues and renal cancer cells, its overexpression should antagonize the malignant behaviors of cancer cells. miR-4521 upregulation (Fig. 4d) resulted in significant reduced in vitro proliferation, migration and invasion capacities of 786-O (Fig. 6a, c) and ACHN cells (Fig. 6b, d). miR-4521 overexpression might affect 786-O and ACHN growths by promoting their apoptosis. The apoptotic rates of miR-4521 overexpressing 786-O and ACHN cells increased by 48.9% and 62.7% (Fig. 6e). This demonstrates that miR-4521 downexpression increases malignant properties of RCC cells, which might contribute to enhanced development and progression of ccRCC patients. This work also showed miR-4521 level was negatively correlated with FAM129A level in ccRCC progression.

FAM129A (Niban or Clorf24) is liked to thyroid cancer^20^, HNSCC^21^ and sporadic renal carcinomas^22^, acting in all as a tumor promoter. FAM129A, ITM1 and PVALB were the three earliest genes used for distinguishing benign thyroid nodules from malignant ones^18–20^. FAM129A was absent in specimens from normal thyroid, benign follicular thyroid adenoma and thyroid hyperplasia, but was present and upregulated in papillary thyroid carcinoma and follicular thyroid carcinoma^20^ tissues. It was consistently found to be more highly expressed in FTC cell lines FTC133, FTC236, FTC238 and WRO, and in the TPC cell line, TPC1, in comparison with a normal thyroid cell line, PCCL3^20^. FAM129A knockdown antagonized the in vitro proliferation, migration and invasion capacities of WRO and TPC1. FAM129 upregulation promoted the carcinogenic process of HNSCC and head and neck dysplastic lesions. Compared with its absence in normal HNS epithelia, IHC assays indicated that FAM129A was positively detected in 42 out of 43 HNSCCs (97.6%) and 20 of 30 (66.6%) dysplastic lesions. FAM129A expression frequently began at HNSCC early stages and continued to be upregulated throughout the carcinogenesis^21^. FAM129A expression was detected in Tsc1 and Tsc2 knockout mice, in sporadic human RCC including clear cell carcinomas, granular cell carcinomas and spindle cell carcinomas. Its common expression might be a marker for renal carcinogenesis^17,22^. We propose that FAM129A acts in renal carcinoma by influencing tumor cell apoptosis based on its involvement in renal interstitial fibrosis via promoting renal tubular cells apoptosis^23^. This work revealed FAM129A as a promotor for ccRCC malignancy. *FAM129A* upregulation (Fig. 2a, b) was positively correlated with advances in TNM stage (Fig. 2c, Table 1) and Fuhrman grade (Fig. 2d, Table 1) of ccRCC patients. Both IHC and WB assays indicated FAM129A protein expression level was increased in patients’ tumorous tissues (Fig. 2e, f, Table 2). The positive immunoreactivity against FAM129A was 70% higher, the positive detection rates of FAM129A with ++ and +++ degrees were 4-fold and 17-fold higher (Table 2), and the overall level determined by WB was 214.7% higher (Fig. 2g, h, Table 2) in patients’ tumorous tissues than in nontumorous tissues. Both IHC and WB assays indicated that FAM129A upregulation was positively correlated with TNM advance (Fig. 2i) and tended to be associated with Fuhrman grade advance (Tables 1 and 2) among patient’s clinicopathological parameters. FAM129A was consistently more highly expressed in renal cancer cells, 786-O and ACHN, than in the normal renal cell, HK-2 (Fig. 4a). Consequently, FAM129A knockdown (Fig. 5a, b) resulted in ameliorated proliferation (Fig. 5c, d), migration and invasion (Fig. 5e, f) capacities of 786-O and ACHN cells. Its downregulation led to increased apoptosis rates of 786-O and ACHN (Fig. 5g), which decreased their growth velocity. FAM129A upregulation aggravates renal carcinoma cell malignancy by promoting ccRCC progression.

miR-4521 deficiency was both inversely correlated with upregulations of FAM129A mRNA and with protein (Fig. 3b, *P* = 0.0006; Fig. 3c, *P* = 0.020) in ccRCC patients’ tumorous specimens. miR-4521 reduction and FAM129A increase in 786-O and ACHN cells than HK-2 cells were also inversely associated (Fig. 4a). Bioinformatic analysis showed the site AGGAAUC of miR-4521 could bind with the site UCCUUAG (3232–3238) in 3′-UTR of *FAM129A* (Fig. 4b). Their direct interaction was validated by a dual-luciferase reporter assay combined with a mutation of the binding site UCCUUAG for *FAM129A* to CGCACAC (Fig. 4c). miR-4521 overexpressions in 786-O and ACHN (Fig. 4d) decreased FAM129A expressions at mRNA (Fig. 4e) and protein (Fig. 4f) levels. As an upstream direct-regulating molecule, miR-4521 retroregulates the expression and functionality of FAM129A. Its suppression is closely related to FAM129A upregulation, which might synergically enhance ccRCC cell’s malignancy and patient’s progression.

**Fig. 8:**
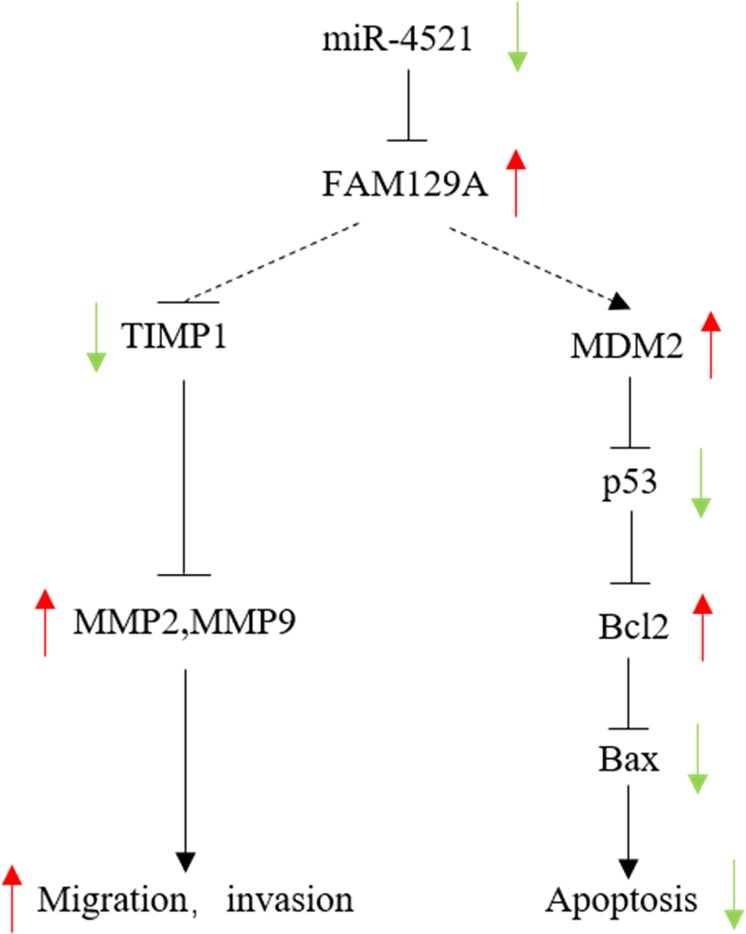
A schematic model of miR-4521-FAM129A axis on ccRCC cell malignant behaviors. miR-4521 induces cell migration and invasion via TIMP-1/ MMP2/MMP9 pathways and also suppresses cell apoptosis by the MDM2/p53/Bcl2/Bax pathway by targeting FAM129A mediating ccRCC malignant behaviors. ccRCC clear cell renal cell carcinoma

Matrix metalloproteinases (MMPs) play important roles in tumor cell proliferation, apoptosis, invasion and metastasis. We investigated changes in the levels of MMP2 and MMP9, two key metalloproteinases, and TIMP1, a key tissue inhibitor of metalloproteinase that regulates most MMPs^28,29^, by following changes in the levels of FAM129A and miR-4521 in RCC cell lines. TIMP-1 was upregulated by 45.3%, and MMP2 and MMP9 were downregulated by 42.4% and 49.2%, respectively, in 786-O cells following FAM129A knockdown (Fig. 7a). These also implicated, acting as a promotor, the upregulation of FAM129A promoted ccRCC progression through enhancing MMP2 and MMP9 activation by suppressing TIMP-1. On the basis of its direct binding to and inverse correlation with FAM129A, we propose miR-4521 should also affect renal cancer cell proliferation, migration and invasion via the above-mentioned molecules. We tried to overexpress and downexpress miR-4521 by mimic transfection and inhibitor transfection in 786-O and ACHN but were unsuccessful. Only miR-4521 overexpression succeeded (Fig. 4d). Its overexpression induced FAM129A downregulation (Figs. 4e, f and 7b), TIMP-1 increased by 68.4%, and MMP2 and MMP9 decreased by 55.1% and 72.5%, respectively (Fig. 7b). As shown in Fig. 8, the above results indicate FAM129A upregulation and/or miR-4521 deficiency contributed FAM129A upregulation affects ccRCC malignancy via the TIMP-1/MMP2/MMP pathway. Interestingly, although FAM129A was more reduced in si-FAM129A-transfected 786-O and ACHN cells (Figs. 4f, 5a, b and 7), more reductions on the invasion and slightly higher reductions on the migration capacities were observed in miR-4521-overexpressing 786-O and ACHN cells (Figs. 5e, f and 6c, d), which was also consistent with more increased TIMP-1 upregulation and MMP2 and MMP9 downregulations resulting in better suppressed invasiveness and metastases of tumor cells. Except for its direct targeting with FAM129A, which has been established, the role of miR-4521 and its detailed action mechanism in ccRCC deserve more attention.

FAM129A upregulation and miR-4521 deficiency are inversely correlated and enhanced cancer cell invasiveness and ccRCC progression (Figs. 1 and 2). FAM129A knockdown and miR-4521 overexpression reduced the proliferations and increased the apoptosis of 786-O and ACHN (Figs. 5 and 6), suggesting that FAM129A overexpression with miR-4521 deficiency might enhance ccRCC progression by promoting cancer cell growth by reducing apoptosis. We showed miR-4521-FAM129A axial regulation in mediating ccRCC progression via MDM2/P53/Bcl-2/Bax. As a tumor suppressor, P53 release from its negative regulator MDM2 induces cell apoptosis^30^ by inhibiting Bcl-2 and enhancing Bax. The Bcl-2/Bax ratio is the critical indictor for apoptosis induced by imbalance of P53 and MDM2 ^30–32^. This work showed that MDM2 and Bcl2 decreased and p53 and Bax increased in 786-O following FAM129A knockdown (Fig. 7a). MDM2 suppression by FAM129A knockdown increased P53 causing Bcl2 reduction and Bax upregulation, which resulted in enhanced apoptosis and decreased proliferation of cancer cells (Figs. 5c, g, 6a, e). As shown in Fig. 8, FAM129A upregulation probably enhances ccRCC malignancy by increasing cancer cell growth through decreased apoptosis induced by p53 suppression. Together, miR-4521 overexpression decreased FAM129A levels in 786-O and ACHN cells (Fig. 4e, f) with decreased proliferations (Fig. 6a, b) and increased apoptosis (Fig. 6e) through MDM2/p53/Bcl2/Bax (Figs. 7b and 8). miR-4521 overexpression led to 40.2% and 50.9% decreases of MDM2 and Bcl2, and 60.6% and 39.5% increases of p53 and Bax, respectively, in 786-O (Fig. 7b), which resulted in an MDM2/P53 imbalance and a Bax/Bcl2 increase for decreased proliferation and enhanced apoptosis of renal cancer cells. miR-4521, negatively regulating FAM129A, functions in ccRCC via MDM2/p53/Bcl2/Bax. Although MDM2 was more decreased (53.1% vs. 40.2%) and p53 was more increased (81.1% vs. 62.7%, Fig. 7) in si-FAM129A knockdown cells, their apoptotic rates were lower than miR-4521-mimic-transfected cells, 42.5% vs. 48.9% (Fig. 5g) and 59.5% vs. 62.7% for 786-O and ACHN group cells (Fig. 6e). Together, miR-4521 overexpression induced lower FAM129A decreases in 786-O and ACHN (Figs. 4e, f, 5a, b and 7), while more decreased cell proliferations were observed for them than the si-FAM129A knockdown group cells, 28.8% vs. 19.0% and 37.4% vs. 12.6%, and 32.1% vs. 22.2% and 28.7% vs. 18.7% (Figs. 5c, d and 6a, b) for 786-O and ACHN groups at 96 and 120 h, respectively. Considering that the Bax/Bcl2 ratio (2.84, 139.5%/49.1%) was higher in miR-4521 overexpressing cells than (2.50, 137.8%/55.0%) in si-FAM129A knockdown cells (Fig. 7), this phenomenon seems reasonable and also suggests the existence of other signaling pathways of miR-4521 in mediating renal cancer cell growth except for MDM2/p53/Bcl2/Bax. Clarification of the role and mechanism of miR-4521 in ccRCC deserves more attention.

Collectively, FAM129A and miR-4521 act as a tumor promotor and suppressor in ccRCC. Negatively correlated, FAM129A upregulation and miR-4521 deficiency contribute to ccRCC clinical progression with enhanced TNM stage and Fuhrman grade. Binding to 3′UTR of *FAM129A*, miR-4521 retroregulates *FAM129A*/FAM129A in mediating ccRCC progression and renal cancer cell malignant properties. FAM129A knockdown or miR-4521 upregulation decreases renal cancer cells’ invasiveness via TIMP-1/MMP2/MMP9 and MDM2/p53/Bcl2/Bax. miR-4521 deficiency with its potential contribution to FAM129A upregulation might synergistically promote renal cancer cells’ malignant behaviors by enhancing MMP2/MMP9 through suppressing TIMP-1 and increasing their growth by escaping apoptosis through suppressing p53 by way of upregulation of MDM2 via a decreased Bax/Bcl2 ratio. The miR-4521-FAM129A axial regulation pathway provides clues useful to the fundamental research, diagnosis and treatment for ccRCC.

## Materials and methods

### Collection and treatment of tumorous and paired paracancerous normal tissues from ccRCC patients

Fifty-five pairs of tumorous and matched paracancerous nontumoral renal tissues from ccRCC patients were collected at the Urology Department of the Second Affiliated Hospital of Dalian Medical University, Dalian. The paracancerous normal renal tissue was taken >5 cm away from the edge of the tumor. No patients received radiotherapy or chemotherapy before surgery. After cleaning, the surgical tissues for western blotting and qRT-PCR assays were immediately snap-frozen in liquid nitrogen and stored at −80 °C. Thirty pairs of sliced surgical tumorous and paracancerous tissues were fixed in 10% neutral-buffered formalin and blocked in paraffin for IHC assay. The ccRCC specimens were classified according to the age, gender, tumor position, TNM stage and Fuhrman grade of patients. The histological subtypes and tumor stages were assessed according to the 2016 WHO Classification of Tumors of the Urinary System of the Tumor, Nodes and Metastasis (TNM) system. Specimen use and investigation protocols were approved by the Medical Ethics Committee of Dalian Medical University. Informed consents were obtained from the patients.

### Quantitative real-time polymerase chain reaction (qRT-PCR) assay

Total RNA was extracted either from each group of specimens or cells by using Trizol™ reagent (Invitrogen, USA) according to the instruction manual. A piece of tissue of ~50 mg was cut into 4 μm slices by a freezing microtome for RNA extraction. Then the EasyScript One-Step gDNA Removal and cDNA Synthesis SuperMix kit (TransGen, China) was used for cDNA reverse transfection. qRT-PCR was carried out on a StepOnePlus™ Real-Time PCR system (ThermoFisher, USA) using FastStart Universal SYBR Green Master (ROX) reagent (Roche, Germany). SnRNA U6 and β-actin were used as internal standards for miR-4521 and *FAM129A*. miR-4521 and U6 primers were purchased from RiboBio Company (Guangzhou, China). Primers were designed as below for *FAM129A*, F: 5′-CTCAGCCCTTTGTGGTCCT-3′, R: 5′-CTC CTGTCGGAAGAATTGCAC-3′ and for β-actin, F: 5′-AGGCCAACCGCGAGAAG-3′, R: 5′-AGAGCCTGGATAGCAACGTACA-3′. The 2^−∆∆CT^ method was performed for quantification analysis.

### Cell culture, miR-4521 overexpression and FAM129 si-RNA interference

Human renal clear cell carcinoma cell lines 786-O and ACHN, and renal proximal tubule cell line HK-2 were used. HK-2, a normal renal cell line, was used to check miR-4521 and FAM129A levels in 786-O and ACHN. Cells were inoculated in RPMI-1640 medium (Gibco, USA) supplemented with 15% fetal bovine serum (FBS, ExCell Bio, China) at 37 °C with 5% CO_2_. The miR-4521 mimic and an irrelevant transfection mimic as NC (negative control) mimic were ordered from GenePharma (Suzhou, China). Si-RNA sequences of *FAM129A*, F: 5′-CCAGCUUAACAGAUCUAAA-3′, R: 5′-UUUAGAUCUGUUAAGCUGG-3′ and of NC si-RNA, F: 5′-UUCUCCGAACGU GUCACGUTT-3′, R: 5′-ACGUGACACGUUCGGAGAATT-3′ were from RiboBio (Guangzhou, China).

For transfection, 786-O and ACHN growing in logarithmic phases were used. Briefly, the densities of 786-O and ACHN cells were adjusted to 5 × 10^4^ cells/mL and 1 × 10^5^ cells/mL using 15% FBS. Two milliliters of each group of 786-O and ACHN cell suspensions were separately distributed in the wells of six-well plate and incubated at 37 °C with 5% CO_2_ for 24 h. Then 4 μL of each of the NC mimic, miR-4521 mimic, si-NC and si-FAM129A with concentration of 20 μM was separately mixed with 50 μL of RPMI-1640 medium, shaken gently and left for 5 min at RT. Simultaneously, 4 μL of lipofectamine™ 2000 (Invitrogen, USA) transfection reagent was added into 50 μL RPMI-1640 medium, shaken gently and left for 5 min at RT. Then the prepared miRNA mimic and siRNA solution was mixed with lipofectamine™ transfection solution, shaken well and left for 20 min at RT. Finally, 100 μL of the above mixture was added into each of the already prepared group cells, gently mixed well, and incubated at 37 °C with 5% CO_2_ for 24 h. The cells from each group were trypsinized with trypsin-EDTA (0.25%, Gibco, USA) and centrifuged at 1000 rpm for 5 min. Cell pellets were collected for further experiments.

### SDS-PAGE and western blotting assay

#### Protein extraction

For protein extraction from specimens, ~50 mg of tumorous or paracancerous tissue was washed with PBS (4 °C), cut into small pieces, ground under liquid nitrogen into powder and suspended in 300–600 μL ice-cold RIPA buffer (50 mM pH 8.0 Tris-HCl, 150 mM NaCl, 1% Triton X-100, 0.5% sodium deoxycholate, 0.1% SDS, 1 mM Na_3_VO_4_, 1 μg/mL leupeptin and 0.5 mM PMSF). The tissue mixture was then ground well with a pestle for 3 × 10 min on ice. Supernatant protein was collected by 12,000 rpm centrifugation at 4 °C for 15 min. For protein extraction from cells, each cell pellet per well obtained from six-well plates of 786-O/ACHN or miRNA mimic-transfected or siRNA-FAM129A-transfected 786-O/ACHN cells was suspended in about 50 μL of the RIPA buffer and lysed on ice for 30 min with suspension using a pipet every 10 min. Supernatant protein was collected by centrifugation at 12,000 rpm at 4 °C for 15 min.

#### Western blotting assay

Protein concentration was determined by the Bradford assay. 10% SDS-PAGE was used for protein separation. Protein bands were transferred onto a nitrocellulose (NC) membrane (PALL, USA), blocked with 5% (w/v) skim milk (BD, USA) in TBST (50 mM Tris, 100 mM NaCl and 0.1% Tween-20, pH 7.5) and incubated with primary antibodies FAM129A (1:1000, Proteintech, USA), GAPDH (1:2000, Proteintech, USA), TIMP1(1:1000, Proteintech, USA), MMP2 (1:500, Proteintech, USA), MMP9 (1:500, Proteintech, USA), MDM2 (1:500, Proteintech, USA), P53 (1:1000, Proteintech, USA), Bcl2 (1:1000, Wanleibio, China) and Bax (1:1500, Proteintech, USA) at 4 °C with shaking at 100 rpm overnight. The NC membrane was washed well with TBST for 3 × 10 min, incubated with HRP-conjugated Affinipure Goat Anti-Rabbit IgG (1:2000, Proteintech, USA) for 2 h at RT, and then washed again with TBST for 3 × 10 min. Protein bands were visualized by ECL and quantified by the Bio-Rad ChemiDoc™ MP system (Bio-Rad, USA).

#### Immunohistochemistry (IHC assay)

The paraffin blocks of tumorous and paracancerous renal tissues were cut into 4-μm slices. The slices were boiled in citrate buffer (0.01 M, pH 6.0) in a microwave oven using high power for 4 min, and cooled to RT. After doing this four times, the slices were washed clean with PBS for 3 × 5 min, blocked in 3% H_2_O_2_ for 20 min and in 10% nonimmune goat serum for 15 min at RT, and incubated with FAM129A antibody (1:200) at 4 °C overnight. The tissue slices were then warmed at 37 °C for 30 min, treated with biotin-streptavidin HRP detection kit (ZSGB-BIO, China), and imaged with 3,3′-diamino-benzidine (DAB) development kit (ZSGB-BIO, China) under an upright light BX3-CBH microscope (Olympus, Japan).

The degree of IHC immunoreactivity was judged by multiplying the staining immunoreaction intensity (Score I) and the DAB positive staining quantity (Score II) of tumor cells as previously reported^33–35^. Score I was classified into four grades, 0 (negative), 1 (weak), 2 (moderate) and 3 (strong). Based on DAB positively stained cells, Score II was rated as 0 (none), 1 (1–10% cells per field), 2 (10–50%), 3 (51–75%) and 4 (>76%). Immunoreactivity degrees with scores of 0–2, 3–5, 6–8 and 9–12 were considered as negative (−), weak (+), moderate (++) and strong (+++) expression of FAM129A. IHC assays were separately scored by two experienced pathologists.

#### Dual-luciferase reporter assay

PsiCHECK™2.0 dual-luciferase expression vector (Promega, USA) was used as expression vector in a dual-luciferase reporter assay as we previously reported^36^. Briefly, one potential binding site of miR-4521 was revealed in the 3′-UTR region of *FAM129A* with the miRDB and TargetScan platforms. The wild-type (WT) and mutant (MUT) binding site sequences in *FAM129*A 3′-UTR were amplified and cloned into psiCHECK™2.0 vectors. The correct recombinant plasmids, validated by nucleotide sequencing (Invitrogen, USA), were named as wt-FAM129A-3′UTR and mut-FAM129A-3′UTR, respectively. 5 × 10^4^ 786-O cells in 2 mL of RPMI-1640 with 15% FBS were seeded into each well of a six-well plate and incubated at 37 °C, 5% CO_2_ for 24 h. Then, 786‐O cells were cotransfected with the additions of 4 μL of miR-4521 mimic/NC mimic (20 μM) and 4 μL (3 μg) of wt-FAM129A-3’UTR/mut-FAM129A-3′UTR at 37 °C with 5% CO_2_ for 24 h, respectively. A cell pellet from each group, obtained by washing with PBS and centrifuging at 1000 rpm for 5 min, was lysed in 200 μL of passive lysis buffer at 4 °C for 15 min. Then 20 μL of supernatant lysate was loaded into a luminometer tube, mixed well with 100 μL of Luciferase Assay ReagentII (LAR II) for Firefly luciferase activity detection, then mixed with 100 μL of Stop & Glo Reagent for Renilla luciferase activity assay using a GloMax fluorescence reader (Promega, USA).

#### MTT assay for cell proliferation

The influences of changes in the levels of miR-4521 and FAM129A on 786-O and ACHN proliferation were determined by MTT assay. The cells from each 786-O group were seeded into a 96-well plate at the density of 5000 cells in 200 μL of RPMI-1640 with 15% FBS per well. ACHN group cells were seeded at the density of 10,000 cells/well. The cells were continuously incubated at 37 °C, 5% CO_2_ for 24, 48, 72, 96 and 120 h, separately, then incubated with 0.5 mg/mL MTT working solution (Sigma, USA) by replacing culture medium at 37 °C, 5% CO_2_ for 4 h in darkness. After the removal of the supernatant, 150 μL DMSO (Sigma, USA) was added into each well to dissolve formazan crystals. The absorbance at 490 nm was measured using a microplate reader (Thermo, USA) for cell density quantification. Triplicate experiments were performed for each assay.

#### Boyden transwell-chamber assay for cell migration and invasion

The 24-well transwell units with 8 mm I.D. polyester membrane with 8 μm pore size polycarbonate filters (Corning, USA) were employed to investigate the influences of miR-4521 upregulation and FAM129A downregulation on the in vitro migration and invasion properties of 786-O and ACHN cells. For migration assays, 600 μL of RPMI-1640 with 15% FBS was loaded into each lower chamber. 10,000 and 2000 cells from each of the 786-O and the ACHN groups were separately loaded into one upper chamber in 200 μL of RPMI-1640 medium and incubated at 37 °C with 5% CO_2_ for 24 h. The cells on the upper surface of the insert that did not migrate were carefully wiped off using cotton swabs. The cells that migrated to the lower surface of the filter were fixed in methanol (AR, Sigma, USA) for 30 min, dried for 5 min at RT, stained in 0.1% crystal violet for 40 min, washed with PBS (200 μL), and counted by randomly selecting five fields per well using an upright light microscope (Olympus, Japan) at a magnification of ×200.

For invasion assays, the filter surface of an insert transwell unit was first coated with 50 μL ice-cold ECM gel (1:5 dilution with RPMI 1640, Sigma, USA) by incubating at 37 °C for 8 h. The loading numbers for the 786-O and the ACHN group cells were 7500 and 15,000, respectively, in 200 μL of RPMI-1640. The remaining steps were the same as for the migration assays.

#### Flow cytometry

Flow cytometry was performed to investigate miR-4521 upregulation and FAM129A downregulation on the apoptosis of 786-O and ACHN cells using the Annexin V Apoptosis Detection Kit APC (Affymetrix eBioscience, USA). Following transfection, 1 × 10^6^ cells from each of 786-O and ACHN groups were harvested, washed once with ice-cold PBS, washed once with the binding buffer, centrifuged at 1000 rpm for 5 min, resuspended in 100 μL binding buffer with the addition of 5 μL of fluorochrome-conjugated Annexin V, and incubated in the dark for 15 min at RT. Finally, the cells from each of 786-O and ACHN groups were washed again with 100 μL binding buffer, resuspended in 200 μL of binding buffer with the addition of 5 μL of propidium iodide (PI) staining solution, and analyzed using a flow cytometer (BD Biosciences, USA). Each assay was replicated for four times.

#### Data processing and statistical analysis

The data were expressed as mean ± SD of at least triplicate independent experiments. SPSS17.0 was used for statistical analysis. The difference between two groups were evaluated by Student’s *t* test and chi-square test analyses. One-way ANOVA analysis was used to evaluate the difference between different groups. Results with *P* < 0.05 were statistically significant.

## Supplementary information

supplemental material
