# Supplementary material for: miR-4521-FAM129A axial regulation on ccRCC progression through TIMP-1/MMP2/MMP9 and MDM2/p53/Bcl2/Bax pathways
Source: Cell Death Discov. 2019 Apr 15;5:89. doi: 10.1038/s41420-019-0167-5 (PMC6465337; doi:10.1038/s41420-019-0167-5)
Supplement: Supplementary file 1 — supplemental material [file 41420_2019_167_MOESM1_ESM.pdf]

DECLARATION OF CONTRIBUTIONS TO ARTICLE

ADMC

Manuscript Number: CDDIS-19-0221	Journal Name: *Cell Death & Disease*	(the 'Journal')
Proposed Title of the Contribution: miR-4521-FAM129A axial regulation on ccRCC progression through TIMP-1/MMP2/MMP9 and MDM2/p53/Bcl2/Bax pathways		(the 'Contribution)
Author(s): Xue Feng, Naimeng Yan, Weibin Sun, Shanliang Zheng, Sixiong Jiang, Jinxia Wang, Chunmei Guo, Lihong Hao, Yuxiang Tian, Shuqing Liu, Ming-Zhong Sun		(the 'Authors')

For all *CDDis* articles, each person named as an author in the published version must be able to show he or she has contributed substantially to the article.

Authorship credit should be based on 1) substantial contributions to conception and design, acquisition of data, or analysis and interpretation of data; 2) drafting the article or revising it critically for important intellectual content; and 3) final approval of the version to be published. Authors should meet conditions 1, 2 and 3.

Any person who cannot be shown to have made a substantial contribution to the article cannot be listed as an author in the final version. The name of any person who is deemed to have made a minor contribution can, however, appear in the Acknowledgments section of the article.

Please complete the table below to indicate the contributions of all named authors to the manuscript.

Author Full Name:	Specification of Contribution to the Manuscript:
Xue Feng	designed the work, performed the experiments , drafted the manuscript.
Naimeng Yan	designed the work, performed the experiments , drafted the manuscript.
Weibin Sun	designed the work and validated the clinical and pathological diagnosis of ccRCC patinets.
Shanliang Zheng	carried out the date analysis and statistics.
Sixiong Jiang	collected and classified the tissues and specimens.
Jinxia Wang	carried out the date analysis and statistics.
Chunmei Guo	carried out the date analysis and statistics.
Lihong Hao	contributed in the IHC image processing and analysis.
Yuxiang Tian	collected and classified the tissues and specimens.
Shuqing Liu	designed the work, revised the manuscript and made the final approval of the submission.
Ming-Zhong Sun	designed the work, revised the manuscript and made the final approval of the submission.

Scanned with CamScanner

Please complete the table below to indicate the contributions of all named authors to the figures.

Figure 1:

Xue Feng and Naimeng Yandesigned the work and performed the experiments, Weibin Sun validated the clinical and pathological diagnosis of ccRCC patinets, Sixiong Jiang and Yuxiang Tian collected and classified the tissues and specimens.Shuqing Liu and Ming-Zhong Sun designed the work and revised the manuscript.

Figure 2:

Xue Feng and Naimeng Yandesigned the work and performed the experiments, Weibin Sun validated the clinical and pathological diagnosis of ccRCC patinets, Sixiong Jiang and Yuxiang Tian collected and classified the tissues and specimens. Lihong Hao contributed in the IHC image processing and analysis.Shuqing Liu and Ming-Zhong Sun designed the work and revised the manuscript.

Figure 3:

Xue Feng and Naimeng Yandesigned the work and performed the experiments, Shanliang Zheng, Jinxia Wang and Chunmei Guo carried out the date analysis and statistics.Shuqing Liu and Ming-Zhong Sun designed the work and revised the manuscript.

Figure 4:

Xue Feng and Naimeng Yandesigned the work and performed the experiments, Shanliang Zheng, Jinxia Wang and Chunmei Guo carried out the date analysis and statistics.Shuqing Liu and Ming-Zhong Sun designed the work and revised the manuscript.

Figure 5:

Xue Feng and Naimeng Yan designed the work and performed the experiments, Shanliang Zheng, Jinxia Wang and Chunmei Guo carried out the date analysis and statistics.Shuqing Liu and Ming-Zhong Sun designed the work and revised the manuscript.

Figure 6:

Xue Feng and Naimeng Yandesigned the work and performed the experiments, Shanliang Zheng, Jinxia Wang and Chunmei Guo carried out the date analysis and statistics.Shuqing Liu and Ming-Zhong Sun designed the work and revised the manuscript.

Signed for and on behalf of the Author(s):	Print Name:	Date:
Ming-zhong Sun	Ming-Zhong Sun	2019-01-24

Scanned with CamScanner
